# Sustained‐Release Sitagliptin Microneedles for Scar Prevention via Fibroblast‐to‐Adipocyte Conversion

**DOI:** 10.1002/smsc.202500140

**Published:** 2025-10-21

**Authors:** Ju‐Lei Zhang, Jun‐Nian Zhou, Chao Tang, Yan Li, Wen‐De Yao, Ling‐Li Guo, Zhao‐Yang Chen, Ya‐Li Jia, Quan Zeng, Biao Zhang, Tao Fan, Jia‐Fei Xi, Xue‐Tao Pei, Yan Han, Wen Yue

**Affiliations:** ^1^ Department of Plastic and Reconstructive Surgery The First Medical Centre Chinese PLA General Hospital 28 Fuxing Street Beijing 100853 China; ^2^ Beijing Institute of Radiation Medicine 27 Taiping Road Beijing 100850 China; ^3^ Department of Plastic Surgery Peking University International Hospital 1 Shengmingyuan Road Beijing 102206 China

**Keywords:** dipeptidyl peptidase IV, hydrogel microneedle patch, keloid, mesenchymal stem cell, sitagliptin

## Abstract

Pathological scar treatment remains a clinical challenge, and novel efficient and safe approaches are urgently needed. Regulation of cell fate transition has promising potential for disease treatment and tissue regeneration. Skin fibrosis is linked to a specific fibroblast subtype marked by dipeptidyl peptidase IV (DPP4^+^), by which various agents, including sitagliptin, an established antidiabetic medication, can inhibit. In this study, it is hypothesized that pharmacological inhibition of DPP4 with sitagliptin could redirect fibroblasts toward adipogenic lineages, consequently, preventing scar formation. Fibroblasts from human keloid tissues are first isolated and characterized, confirming their mesenchymal stem cell (MSCs) properties and termed them as keloid‐derived MSCs (KMSCs). The analyses reveal that DPP4^−^ KMSCs exhibit enhanced adipogenic potential, whereas DPP4^+^ KMSCs display greater fibrotic potential. In KMSCs, sitagliptin promotes adipogenesis by inhibiting DPP4‐mediated IGF1 truncation, thereby enhancing IGF1 signaling. Furthermore, sitagliptin‐loaded microneedle patches are developed capable of sustained, controlled release of sitagliptin or IGF1 into cutaneous wounds, effectively reducing scar formation by promoting the conversion of fibroblasts into adipocytes in vivo. Overall, the findings propose a novel application for sitagliptin in preventing scar formation via cell fate modulation during wound healing, thereby advancing clinical treatment strategies for scars.

## Graphical abstract

The authors propose a novel use for the conventional hypoglycemic drug, dipeptidyl peptidase IV (DPP4^+^) inhibitor sitagliptin. Sitagliptin prevents scar formation during wound healing through fibroblast‐to‐adipocyte conversion at least by preventing DPP4‐mediated truncation of IGF‐1. This holds great promise to enable “scarless healing”, addressing an unmet need in the field of regenerative medicine and wound management.

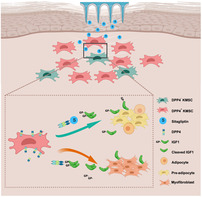

## 1 Introduction

Hypertrophic scars and keloids frequently result in significant cosmetic concerns, abnormal sensations, functional impairment, and even pose a risk of carcinogenesis,^[^ ^1^ ^]^ resulting in a huge burden to patients and society.^[^ ^2^, ^3^, ^4^ ^]^ However, current approaches to scar prevention and treatment remain inadequate and are often associated with high rates of recurrence.^[^ ^5^, ^6^ ^]^ Studies at the cellular subpopulation level have elucidated the wound healing and scar formation process, facilitating improved control of the healing procedure to achieve better results.

Fibroblasts and myofibroblasts serve as the principal effector cells in wound healing by synthesizing extracellular matrix (ECM) components and mediating wound contraction to facilitate tissue repair. Efforts are currently underway to clarify the fibrotic process in order to develop new strategies for scar prevention. Longaker and colleagues demonstrated the presence of two distinct fibroblast lineages responsible for skin regeneration and scar formation. Notably, these two lineages can be discriminated by the expression of dipeptidyl peptidase IV(DPP4)^[^ ^7^ ^]^ with DPP4^+^ fibroblasts identified as key mediators of cutaneous fibrosis.^[^ ^8^, ^9^, ^10^ ^]^ Emerging evidence indicates that adipocytes, preadipocytes, or lipofibroblasts actively participate in wound healing and scar formation,^[^ ^11^, ^12^ ^]^ and that myofibroblasts can transform into adipocytes, suggesting an intrinsic link between fibrosis and adipogenesis.^[^ ^13^ ^]^ Importantly, DPP4 expression declines during the differentiation of mesenchymal progenitor cells into committed preadipocytes;^[^ ^14^ ^]^ furthermore, DPP4 knockdown inhibits preadipocyte proliferation and enhances adipocyte maturation,^[^ ^15^ ^]^ suggesting that DPP4 inhibition may promote adipogenic differentiation and suppress the fibrotic process.

With advancements in research on cell plasticity and lineage reprogramming, regulation of cell fate transition has emerged as a promising strategy for reversing diseases and promoting tissue regeneration.^[^ ^16^, ^17^ ^]^ Unlike conventional cytotoxic therapies for fibrosis that nonselectively target fibroblasts, cell fate conversion approaches target disease pathogenesis by directing cellular differentiation or redirection. These strategies have two major advantages: 1) inflammatory responses caused by secondary apoptosis or necrosis are avoided and 2) wound regeneration, including subcutaneous adipose tissue restoration, is actively promoted to achieve perfect healing.^[^ ^13^, ^18^, ^19^ ^]^

DPP4 is a key regulator of blood glucose homeostasis due to its enzymatic activity.^[^ ^20^ ^]^ Sitagliptin, a clinically utilized DPP4 inhibitor, is widely used for glycemic control.^[^ ^20^, ^21^, ^22^ ^]^ Here, we hypothesized that pharmacological inhibition of DPP4 using sitagliptin could induce the conversion of fibroblasts into adipocytes, thereby preventing scar formation.

In this study, we first evaluated the distinctive functions of DPP4^+^ and DPP4^−^ fibroblastic cells from human keloids, assessing their potential for multidirectional differentiation. Our results showed that DPP4^−^ keloid‐derived mesenchymal stem cell (KMSC) has a higher adipogenic potential both in vitro and in vivo. The inhibition of DPP4 activity by sitagliptin reprogrammed keloid‐derived fibroblastic cells toward an adipogenic fate through protection of insulin‐like growth factor 1 (IGF1) activity. Building upon established microneedle technology,^[^ ^23^, ^24^, ^25^ ^]^ we developed an effective transdermal delivery system composed of hyaluronic acid methacrylate (HAMA) microneedle patches (MNPs), to provide sustained and controlled release of sitagliptin into skin wounds. To our knowledge, our findings represent the first time that MNPs loaded with sitagliptin or IGF1 can effectively inhibit scar formation by directing cell fate conversion.

## 2 Results

### 2.1 DPP4^−^ KMSCs Exhibit a Higher Adipogenic Potential In Vitro

**Figure 1:**
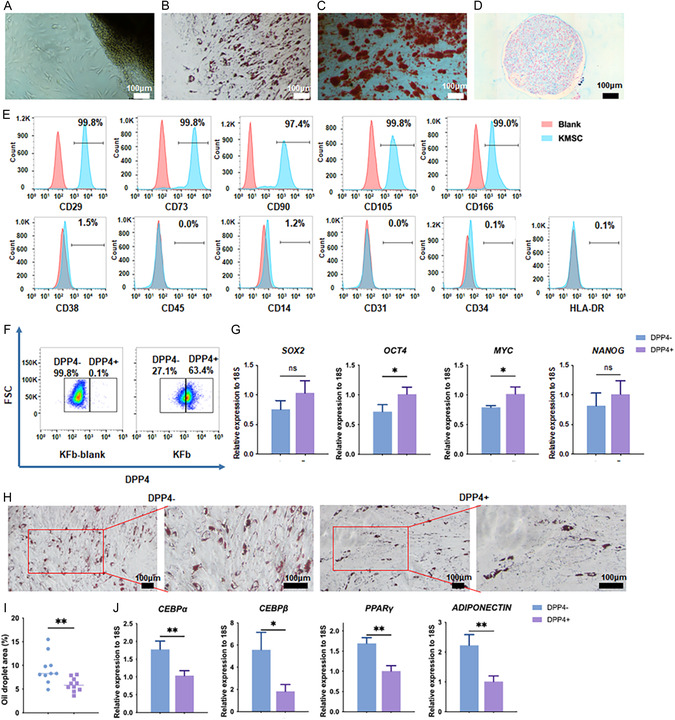
DPP4^−^ KMSCs exhibit higher adipogenic potential in vitro. A–D) Keloid tissues were collected, minced, and seeded to isolate and culture keloid‐derived fibroblasts (A). The isolated fibroblasts were then differentiated toward adipogenic (B), osteogenic (C), and chondrogenic fates (D). E) Surface marker expressions were analyzed using flow cytometry. F) Keloid‐derived DPP4^+^ and DPP4^−^ fibroblasts were sorted via fluorescence‐activated cell sorting. G) RT‐qPCR was performed to evaluate the mRNA level of *OCT4*, *MYC*, *SOX2*, and *NANOG*. n = 3; *, *p *< 0.05. H–J) Sorted DPP4^+/−^ fibroblasts underwent adipogenic induction, followed by Oil Red O staining and quantification analysis (H–I). Ten random fields per sample were analyzed. The mRNA levels of adipogenesis‐related genes were detected using RT‐qPCR (J); n = 3. Scale bars, 100 μm in (A, B, C, D, and H). Data are presented as mean ± SEM; statistical significance was determined by Student's *t*‐test.

Keloid‐derived fibroblasts were isolated and cultured as previously described (**Figure** **1**A).^[^ ^26^ ^]^ To assess the multipotent differentiation capacity of these cells, we subjected them to standardized trilineage induction protocols used for mesenchymal stem cells (MSCs). Our results demonstrated that these cells could differentiate into adipocyte‐like, osteoblast‐like, and chondrocyte‐like cells, as evidenced by the accumulation of small lipid droplets (Figure 1B), deposition of a calcium‐rich mineralized matrix (Figure 1C), and production of acidic mucopolysaccharides (Figure 1D). Flow cytometry confirmed high expression of canonical MSC markers—CD29, CD73, CD90, CD105, and CD166—while excluding markers for hematopoietic, immune, and endothelial lineages (CD34, CD38, CD45, CD14, HLA‐DR, and CD31)^[^ ^27^ ^]^ (Figure 1E). These results confirmed that fibroblasts from human keloids possess defining MSC characteristics, and we accordingly termed them as KMSCs.

Given that DPP4 serves as a marker distinguishing fibroblast subpopulations,^[^ ^7^ ^]^ we examined whether DPP4 expression defines functionally distinct KMSC subsets. Flow cytometry analysis revealed that 63.4% of the KMSCs were DPP4‐positive (Figure 1F). To further explore functional differences, we sorted DPP4^+^ and DPP4^−^ KMSCs for further analysis. The colony‐forming unit‐fibroblast (CFU‐F) assay did not show any difference in clonogenicity between the two groups (Figure S1, Supporting Information). RT‐qPCR analysis revealed modest differences in the expression levels of stemness‐associated genes: DPP4^+^ KMSCs expressed higher levels of OCT4 and MYC compared to DPP4^−^ KMSCs, while the levels of SOX2 and NANOG remained similar (Figure 1G).

Next, to compare the differentiation potential of DPP4^+^ and DPP4^−^ KMSCs, these cells were cultured in media for trilineage differentiation. Notably, the DPP4^−^ KMSCs showed a higher oil‐droplet‐forming area in adipogenic induction medium (Figure 1H–I), as well as increased expression of adipogenic markers—CCAAT/enhancer‐binding protein alpha (*CEBPα*), CCAAT/enhancer‐binding protein beta (*CEBPβ*), peroxisome proliferator‐activated receptor gamma (*PPARγ*), and *ADIPONECTIN*, compared with those in the same number of DPP4^+^ KMSCs (Figure 1J). Formation of a calcium‐rich mineralized matrix (Figure S2A–B, Supporting Information) and acidic mucopolysaccharides (Figure S2D–E, Supporting Information), as well as the expression of osteogenic and chondrogenic genes (Figure S2C,F, Supporting Information), did not differ significantly between each group, indicating the similar osteogenic and chondrogenic potential. These results suggested DPP4^−^ KMSCs have a higher adipogenic potential than that of DPP4^+^ KMSCs in vitro.

### 2.2 DPP4^−^ KMSCs Display Increased Adipogenic Potential, while DPP4^+^ KMSCs Exhibit Higher Fibrotic Potential In Vivo

**Figure 2:**
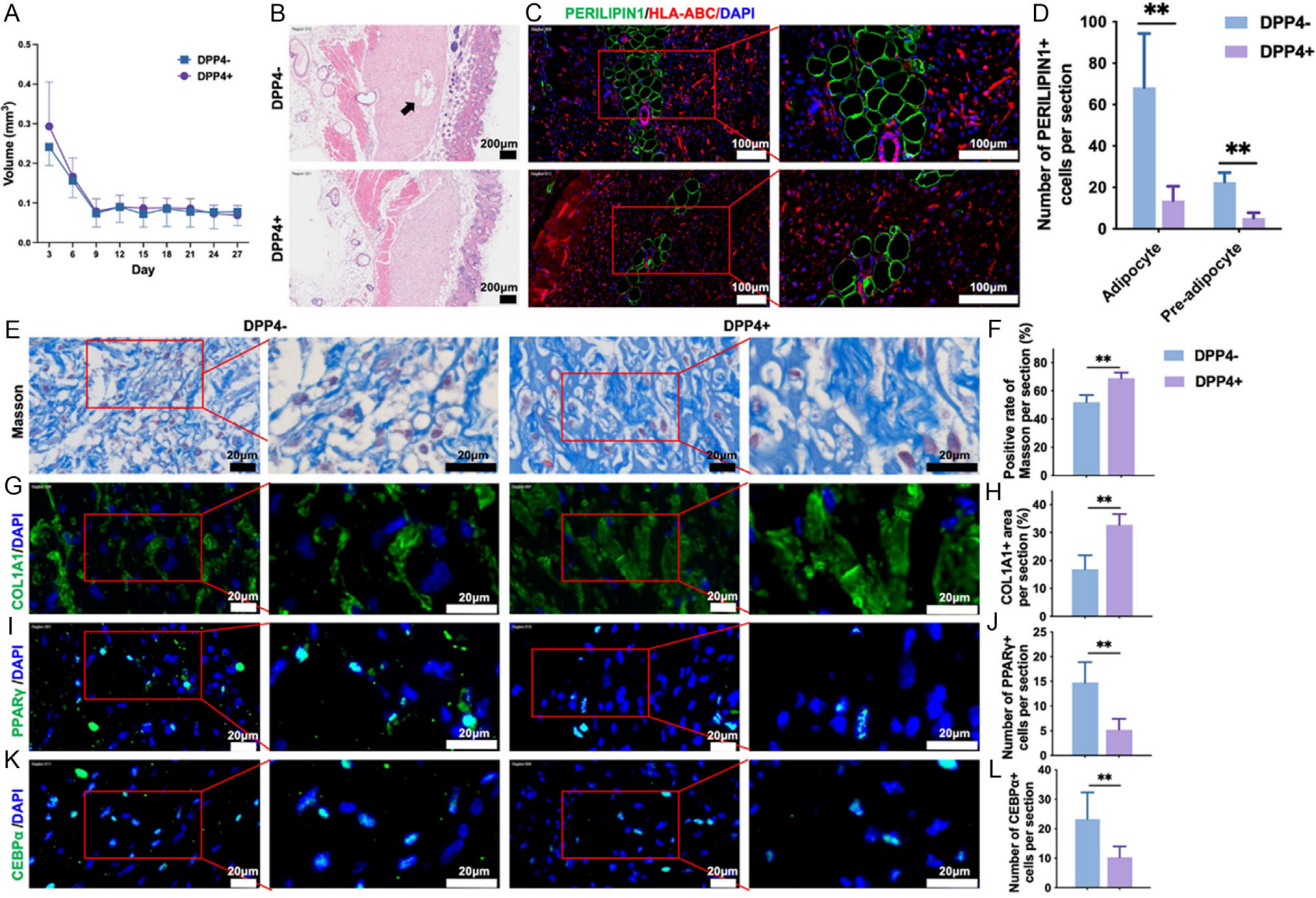
DPP4^−^ KMSCs demonstrate enhanced adipogenic capacity, whereas DPP4^+^ KMSCs demonstrate increased fibrotic capacity in vivo. FACS‐sorted DPP4^+^ and DPP4^−^ cells were subcutaneously transplanted into Balb/c nude mice. A) The volume of subcutaneously transplanted KMSCs in mice was measured, n = 9. B–D) Adipocyte formation was evaluated by (B) H&E staining (black arrow) and (C) immunofluorescence staining of Perilipin and HLA‐ABC. The black arrow indicates the newly formed adipocytes. (D) The numbers of adipocytes and immature adipocytes were analyzed; n = 6. E,F) Masson's trichrome staining and G,H) immunofluorescence staining of COL1A1 were performed to evaluate collagen deposition. The expression of I,J) PPARγ and K,L) CEBPα was examined by immunofluorescence staining. n = 6. **, *p *< 0.01. Scale bars, 200 μm in (B), 100 μm in (C), 20 μm in (E, G, I, and K). Data were presented as mean ± SEM. Statistical significance was determined by Student's *t*‐test.

To further verify the distinct adipogenic capacities of DPP4^+^ and DPP4^−^ KMSCs in vivo, sorted cell populations were transplanted subcutaneously into Balb/c nude mice, with xenograft volumes monitored over four weeks. Both groups initially exhibited a decrease in xenograft size, followed by the steady stage. No significant difference in the xenograft volume was observed between groups (**Figure** **2**A), indicating DPP4^+^ and DPP4^−^ KMSCs have the same growth capacity in vivo. The adipogenic capacity of the transplanted KMSCs was then evaluated (Figure 2B–C). In mice receiving DPP4^−^ KMSCs, the transplanted zone showed more adipocytes (the rounded, vacuolated fat chambers, PERILIPIN1^+^, *p* < 0.01) compared with those in the mice receiving DPP4^+^ KMSCs (Figure 2B–C); similarly, the number of immature adipocytes (preadipocyte, identified by PERILIPIN1^+^ cells without vacuolated fat chambers) was also higher in the DPP4^−^ KMSC group (*p* < 0.01, Figure 2C–D).

As adipocytes are one of the intrinsic components of skin tissues,^[^ ^19^ ^]^ and the transformation from myofibroblasts to adipocytes is a sign of cutaneous regeneration and scar relieving,^[^ ^13^ ^]^ we examined whether this was coupled to reduced fibrosis in the DPP4^−^ KMSC group. Masson staining showed that collagen deposition was significantly alleviated in the DPP4^−^ KMSC group, as indicated by the lower positivity rate of Masson staining and thinner collagen bundles (Figure 2E–F). Similar findings were observed in the immunofluorescence staining of Collagen Type I Alpha 1 Chain (COL1A1) (Figure 2G–H), consistent with the findings of Longaker et al.^[^ ^7^ ^]^ and other groups,^[^ ^8^, ^9^, ^10^ ^]^ demonstrating that DPP4^+^ fibroblasts are the main source of collagen deposition during wound healing and other fibrotic processes. PPARγ and CEBPα are key transcriptional activators of numerous genes related to adipogenesis,^[^ ^28^ ^]^ serving as indicators of adipogenic activity. Immunofluorescence results indicated significantly higher expression levels of PPARγ and CEBPα in the DPP4^−^ group compared to those in the DPP4^+^ group (Figure 2I–L), suggesting a higher adipogenic potential of DPP4^−^ KMSCs in vivo.

Together, these results suggest that DPP4^−^ KMSCs possess a higher adipogenic capacity, while DPP4^+^ KMSC exhibit higher fibrotic capacity in vivo, highlighting DPP4 as a promising target for scar prevention or treatment.

### 2.3 Sitagliptin Enhances Adipogenic Differentiation and Inhibits the Fibrotic Potential in KMSCs

**Figure 3:**
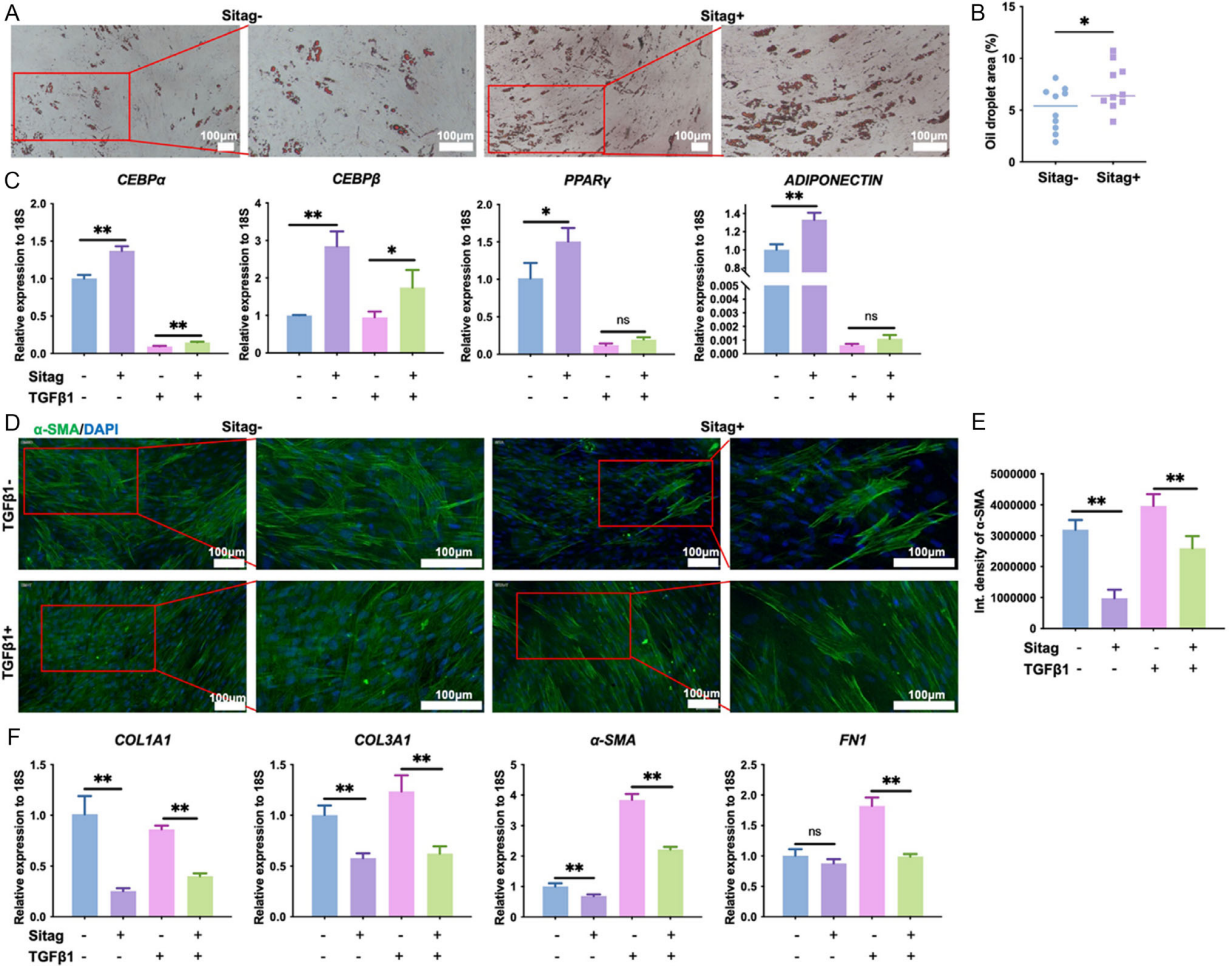
Sitagliptin enhances the adipogenic differentiation and inhibits the profibrotic activity in KMSCs. A–C) KMSCs subjected to adipogenic induction and treated with sitagliptin (20 μM) or TGF‐β1 (10 ng ml^−1^); A,B) lipid droplet formation was then evaluated by Oil Red O staining and quantification analysis. Ten fields of view were randomly selected in each group for analysis. C) The mRNA levels of adipogenesis‐related genes were detected using RT‐qPCR; n = 3. D–F) KMSCs were treated with sitagliptin or TGF‐β1 in DMEM, and D,E) the expression of α‐SMA was evaluated by immunofluorescence staining after 72 h stimulation; n = 5. The mRNA levels of fibrosis‐related genes were determined using RT‐qPCR after 24 h stimulation (F; n = 3). *, *p *< 0.05, **, *p *< 0.01. Scale bars, 100 μm in (A) and 100 μm in (D). Sitag, sitagliptin. Data was presented as mean ± SEM. Statistical significance was determined by one‐way analysis of variance (ANOVA) followed by Tukey's HSD post hoc test.

DPP4 possesses serine peptidase activity, governing the cleavage of glycemic peptides such as glucagon‐like peptide 1 (GLP‐1) and glucose‐dependent insulinotropic polypeptide (GIP), to regulate glycemia.^[^ ^20^ ^]^ DPP4 is also involved in cellular activity in a nonenzymatic manner.^[^ ^29^ ^]^ To explore the functional role of DPP4 in fibrotic and adipogenic processes, we treated cultured KMSCs with sitagliptin, a clinically established DPP4 inhibitor.^[^ ^21^, ^22^ ^]^ The concentration of sitagliptin was optimized based on the effects on PPARγ and α‐SMA protein levels, and the IC_50_ value of sitagliptin (Figures S3,4, Supporting Information). During adipogenic induction, inhibition of DPP4 activity by sitagliptin significantly increased lipid droplet formation (**Figure** **3**A–B) and upregulated mRNA levels of key adipogenic genes (*CEBPα*, *CEBPβ*, *PPARγ,* and *ADIPONECTIN*; Figure 3C). TGF‐β1 stimulation is regarded as an effective in vitro model for fibrosis.^[^ ^30^, ^31^ ^]^ The dose‐dependent effects of TGF‐β1 on KMSC viability were determined with the CCK8 assay (Figure S5, Supporting Information). In TGF‐β1‐treated model, DPP4 inhibition by sitagliptin also promoted the expression of adipogenic genes in a manner similar to that without TGF‐β1‐treatment (Figure 3C). In contrast, DPP4 inhibition suppressed the expression of the myofibroblast/fibrosis marker, α‐SMA (Figure 3D–E), as well as fibrotic genes including COL1A1, Collagen Type III Alpha 1 Chain (COL3A1), and Fibronectin 1 (FN1) (Figure 3F). These data suggest that DPP4 activity in KMSCs, which can be inhibited by sitagliptin, may be a contributing factor to the decreased adipogenesis and increased fibrosis. Collectively, these results suggest that the DPP4 inhibitor sitagliptin enhances adipogenic differentiation and inhibit the fibrotic capacity in KMSCs.

### 2.4 Sitagliptin Promotes Adipogenesis at Least in Part by Protecting IGF1 from DPP4‐Mediated Truncation

**Figure 4:**
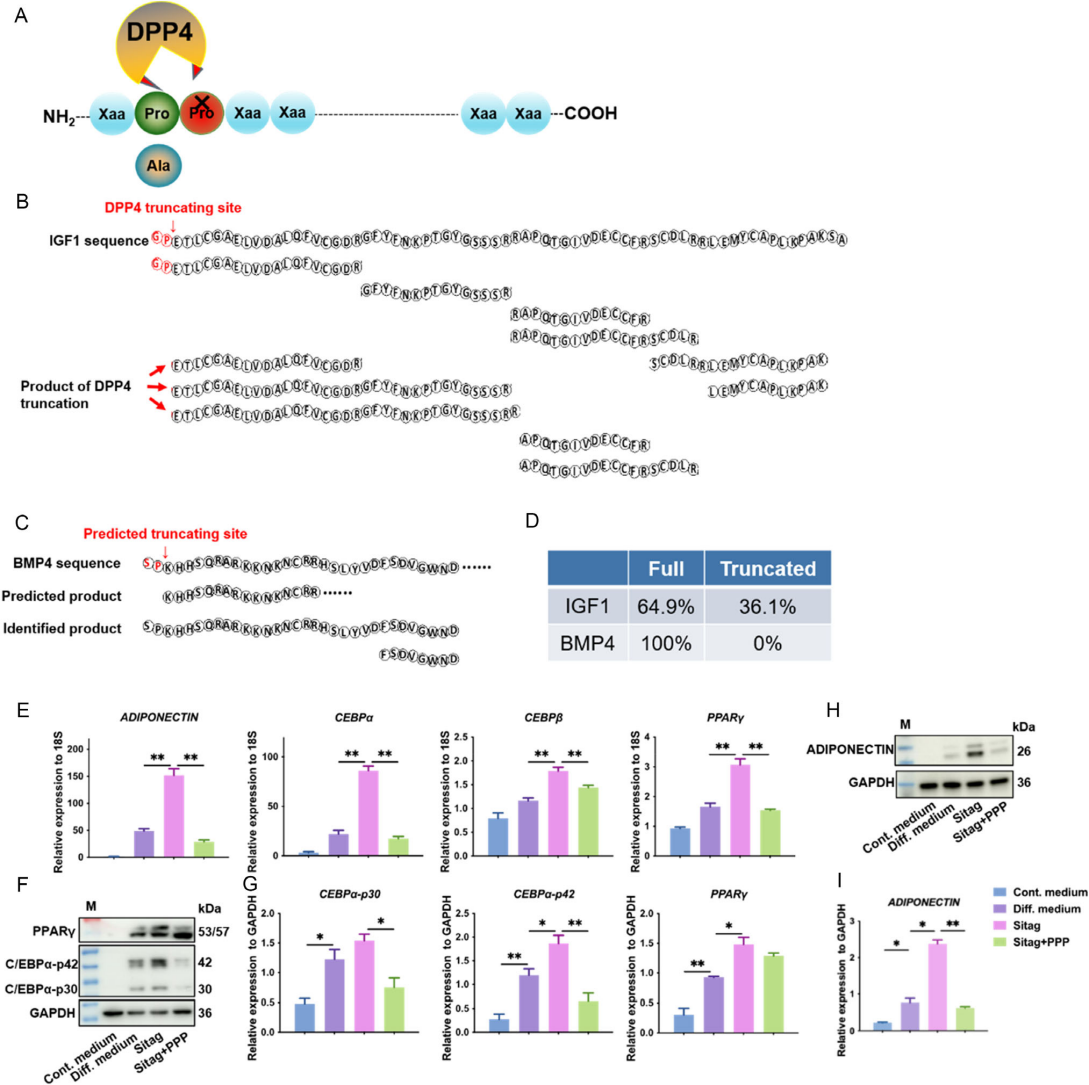
Sitagliptin‐mediated adipogenic promotion is dependent on protection of IGF1 from DPP4 cleavage. A) Schematic of DPP4‐driven peptide/protein truncation. B–D) DPP4 directly truncates IGF1 but not BMP4. Recombinant human IGF‐I or BMP4 was incubated with recombinant human DPP4 Fc chimera for the cleaving assay, and the product was analyzed using HPLC‐MS/MS. (B) The peptide starting with “ETLC” (red arrow) represented the truncated product of IGF1 by DPP4 and (C) peptide starting with “KHHS” represented the truncated product of BMP4 by DPP4. (D) Percentages of truncated product from the cleaving assay were calculated. E–I) KMSCs underwent adipogenic induction and treated with sitagliptin (20 μM) or combined with the IGF‐1 signaling pathway inhibitor picropodophyllin (PPP, 50 nM), the mRNA and protein levels of adipogenesis‐related molecules were detected by RT‐qPCR E) and F–I) western blotting and quantification analysis. n = 3; *, *p *< 0.05, **, *p *< 0.01. Cont. medium, control medium, diff. medium, differentiation medium. Sitag, sitagliptin. M, marker. Data was presented as mean ± SEM. Statistical significance was determined by one‐way analysis of variance (ANOVA) followed by Tukey's HSD post hoc test.

To clarify the mechanisms by which DPP4 and sitagliptin affect adipogenesis and fibrosis, a review of literature on DPP4 substrates was performed. Several proteins are known to be substrates of DPP4, including glucagon‐like peptide 1 (GLP‐1), GIP, peptide YY (PYY), neuropeptide Y (NPY), stromal cell‐derived factor 1 (SDF‐1), vasoactive intestinal polypeptide (VIP), pituitary adenylate cyclase‐activating polypeptide (PACAP), and IGF1.^[^ ^32^ ^]^ Among these substrates, IGF1 was reported to strongly promote adipogenesis in human MSCs.^[^ ^33^ ^]^ To assess DPP4's ability to truncate IGF1, we performed a cleaving assay by incubating recombinant human IGF1 with recombinant human DPP4 Fc chimera. The cleaving products were subsequently analyzed by high‐performance liquid chromatography‐tandem mass spectrometry (HPLC‐MS/MS) and the truncated peptide sequences were analyzed. Previous reports indicate DPP4 cleaves the first two N‐terminal residues when the second amino acid is proline or alanine;^[^ ^34^ ^]^ accordingly, a peptide starting with “ETLC” represented the truncated product of IGF1 generated by DPP4 (**Figure** **4**A,B,D, Figure S6, Supporting Information). We also investigated bone morphogenetic protein 4 (BMP4)—another predicted DPP4 substrate based on sequence homology,^[^ ^28^, ^34^ ^]^ and found that BMP4 was resistant to DPP4 cleavage (Figure 4C–D, Figure S6, Supporting Information). Thus, DPP4 selectively truncates IGF1. Functional analyses by RT‐qPCR and western blot further showed that sitagliptin significantly promoted the expression of adipogenesis‐related genes and proteins including ADIPONECTIN, CEBPα, CEBPβ, and PPARγ. Most importantly, when IGF1 signaling was inhibited with picropodophyllin (PPP),^[^ ^35^ ^]^ the effects of sitagliptin were abolished (Figure 4E–I). Taken together, these results suggest that sitagliptin promotes adipogenesis, at least in part, by preventing DPP4‐mediated IGF1 truncation and preserving IGF1 signaling activity.

### 2.5 Preparation and Characterization of Hydrogel MNPs Loaded with Sitagliptin or IGF1

**Figure 5:**
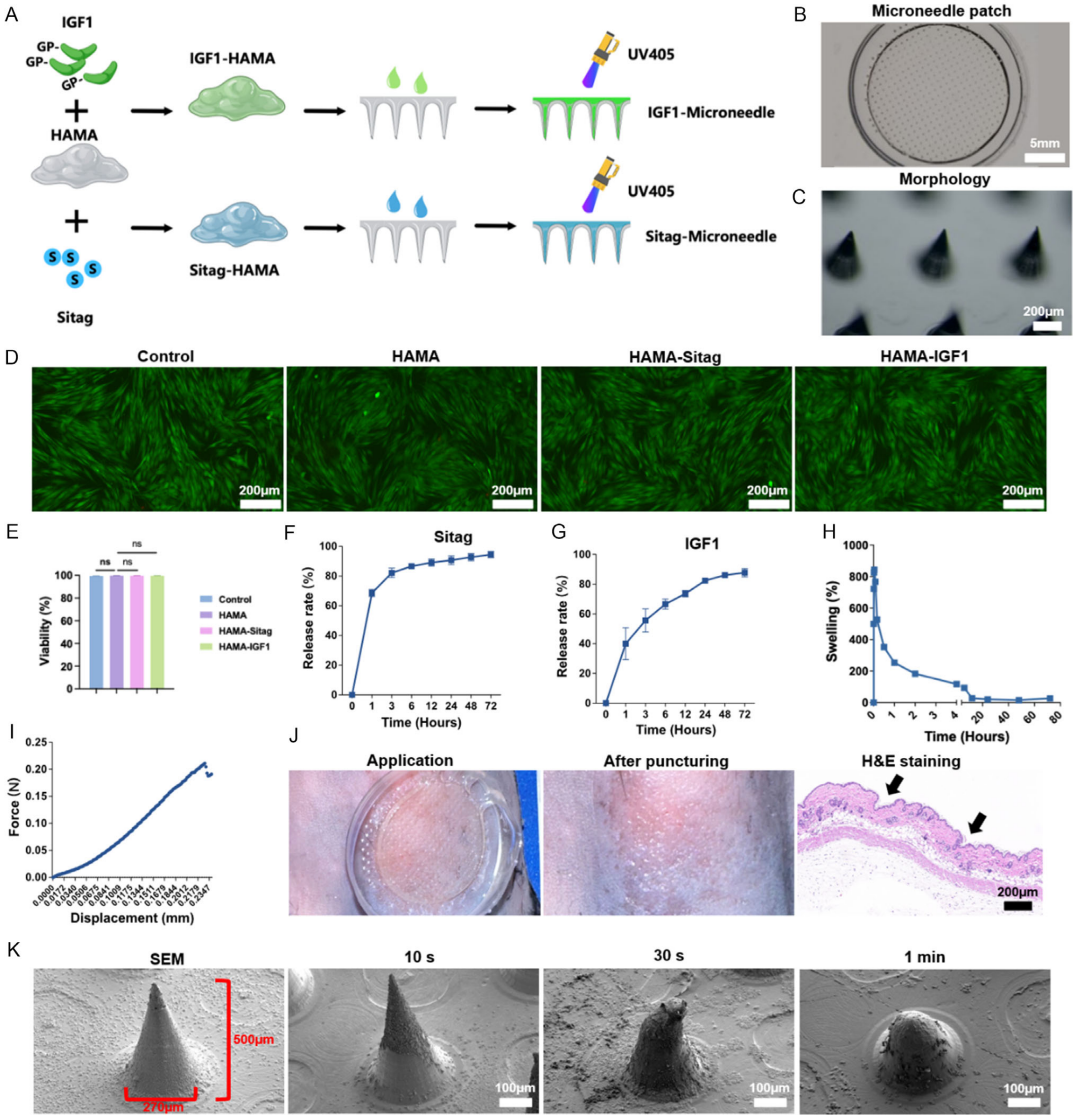
Preparation and characterization of HAMA hydrogel MNPs loaded with sitagliptin or IGF1. A) Schema of MNP preparation. Sitagliptin phosphate monohydrate (2 mg mL^−1^) or IGF1 (500 ng mL^−1^) dissolved in HAMA precursor solutions, formed in a microneedle mold and photocured with UV405 nm light. B,C) Macroscopic and microscopic morphology before and after application. D,E) Live/dead assay was performed and analyzed to evaluate in vitro biocompatibility, n = 3. F,G) Release experiments were performed to investigate the release kinectics of sitagliptin (F) and IGF1 (G; n = 3). H) Swelling property were evaluated, n = 3. I) Compression modulus measurement. J,K) MNPs was pressed into the dorsal skin of C57BL/6 mice, (J) the H&E staining was performed to confirm the penetration, and (K) the surface morphology of the microneedle was observed using SEM. Scale bars, 5 mm in B, 200 μm in (C,D), 200 μm in (J), and 100 μm in (K). Sitag, sitagliptin. HAMA, hyaluronic acid methacrylate. Data was presented as mean ± SEM. Statistical significance was determined by one‐way analysis of variance (ANOVA) followed by Tukey's HSD post hoc test.

To investigate whether IGF1 signaling enhancement via sitagliptin or IGF1 could prevent scar formation during wound healing, we established a hydrogel MNP system for sustained, localized delivery of these agents into the wound bed, as recently reported.^[^ ^36^ ^]^ Briefly, sitagliptin phosphate monohydrate or IGF1 was dissolved into HAMA precursor solution, formed in microneedle molds, and photopolymerized under UV405 nm light, respectively (**Figure** **5**A). The fabricated MNPs displayed a circular appearance with tapered microneedle arrays under the stereomicroscope (Figure 5B–C). There is not any significant difference in cell viability between the control, empty MNP, sitagliptin‐loaded, and IGF1‐loaded groups (Figure 5D–E). Release kinetics results revealed that sitagliptin underwent an initial burst release (≈70% of the total loading dose within the first hour), followed by slower, sustained release reaching 90% by 72 h (Figure 5F), whereas IGF1 was more gradually released (40% at 1 h, 80% at 72 h; Figure 5G). The swelling assay showed that the microneedle weight increased rapidly at the beginning, followed by a gradually decrease until the MNPs dissolved (Figure 5H). Compression modulus tests confirmed that the MNPs possessed adequate mechanical rigidity (0.21 N) to penetrate murine skin (less than 0.1 N^[^ ^37^, ^38^ ^]^) (Figure 5I). In the skin puncture experiments, micropores were observed on the mouse skin after removal of the MNP, verified by hematoxylin and eosin (H&E) staining, confirming the penetration of the microneedles into the skin (Figure 5J). Scan electron microscope (SEM) analysis further indicated partial dissolution and tip blunting after one minute of skin insertion (Figure 5K). These findings demonstrated that sitagliptin or IGF1 loaded MNPs were successfully prepared with robust penetration, controlled release, good biocompatibility, and appropriate degradability.

### 2.6 Sitagliptin or IGF1‐Loaded MNPs Alleviated Scar Formation in the Cutaneous Injury in Mice

**Figure 6:**
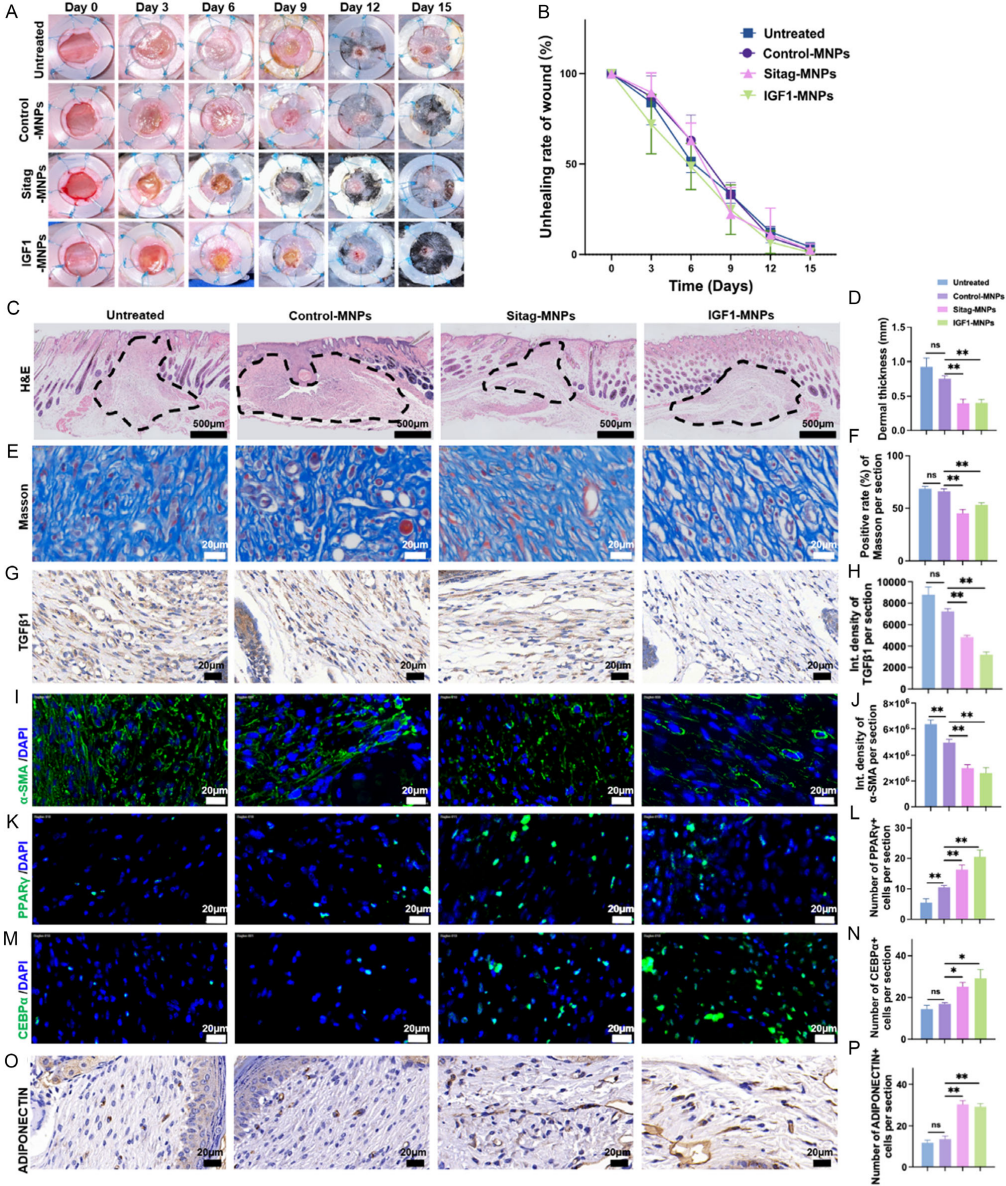
Sitagliptin or IGF1‐loaded MNPs alleviates scar formation, follow cutaneous injury in mice. Full‐thickness dorsal wound model with silicone ring fixation in C57BL/6 mice. A) The healing process was recorded and B) the unhealed wound area was analyzed. C,D) H&E staining was performed and the thickness of the healed dermis (marked with dotted line) was measured, Scale bars, 500 μm in (C). E,F) Masson's trichrome staining, G,H) immunohistochemistry staining of TGFβ1, and I,J) immunofluorescence staining of α‐SMA were performed to evaluate the scar formation. K‐L) The expression of PPARγ and M,N) CEBPα was detected by immunofluorescence staining. O,P) The expression of ADIPONECTIN was detected by immunohistochemistry staining. n = 6; *, *p* < 0.05, **, *p* < 0.01. Scale bars, 20 μm in E, G, I, K, M, and O. MNPs, microneedle patches. Sitag, sitagliptin. Data was presented as mean ± SEM. Statistical significance was determined by one‐way analysis of variance (ANOVA) followed by Tukey's HSD post hoc test.

Finally, a cutaneous scar model was established by generating a full‐thickness dorsal wounds in C57BL/6 mice, maintained with a silicone ring to prevent contraction. Mice were randomly divided into four groups: the untreated, control MNPs (empty), sitagliptin‐loaded MNPs, and IGF1‐loaded MNPs group (**Figure** **6**A; Figure S7, Supporting Information)). During the 15‐day‐healing period, wound area analysis demonstrated similar healing kinetics across each group (Figure 6B), while at endpoint, dermal thickness was significantly decreased in both sitagliptin or IGF1‐loaded MNP groups compared to controls (Figure 6C–D), indicating alleviated scar formation. Masson's trichrome staining analysis demonstrated attenuated collagen deposition and thinner bundles in treated wounds (Figure 6E–F). Moreover, consistent with the in vitro results (Figure 3, Figure 4E–I), the expression levels of TGFβ1 (Figure 6G–H) and α‐SMA (Figure 6I–J) were significantly decreased in wounds treatment with sitagliptin or IGF1‐loaded MNPs. The number of PPARγ^+^ (Figure 6K–L), CEBPα^+^ cells (Figure 6M–N), and ADIPONECTIN^+^ cells (Figure 6O–P) were significantly increased in the treatment groups of sitagliptin or IGF1‐loaded MNPs, respectively, demonstrating the effective inhibition of fibrosis and promotion of adipogenesis at wound sites. CD36, which has pleotropic effects, including lipid metabolism and scar‐related inflammation,^[^ ^12^, ^39^ ^]^ was also examined and no significant changes were observed between the groups (Figure S8, Supporting Information). This might indicate that the efficacy of sitagliptin treatment is independent of alterations in CD36 expression.

Together, these data indicate that by reprogramming myofibroblasts into adipogenic lineages, sitagliptin or IGF1 loaded MNPs effectively inhibit myofibroblast differentiation and alleviate scar formation.

## 3 Discussion

Skin regeneration is defined as restoration of an intact epidermis, dermis, appendage structures, and normal ECM components.^[^ ^40^, ^41^ ^]^ Pathological scars, such as keloids and hypertrophic scars, are characterized by excessive ECM deposition, a disordered epidermis, and the absence of hair follicles and sweat glands. Notably, fat tissue is missing from the scar tissue, as shown by loss of the fat dome in the hypertrophic scar tissue.^[^ ^2^ ^]^ In 2017, Plikus et al. found that in a model of wound‐induced hair neogenesis, myofibroblasts might be reprogrammed and differentiated into adipocytes,^[^ ^13^ ^]^ whereas Brett et al. found that during wound healing, dermal adipocytes underwent lipolysis and a fate switch into myofibroblasts to exert their repair activity.^[^ ^11^ ^]^ In this study, we found that the fibrotic process could be converted into an adipogenic process to alleviate scar formation in mice using sitagliptin or IGF1 treatment, and proved that manipulating cell fate conversion could be a potential treatment for scar prevention.

In addition to our previous experience in isolating MSCs from human cancer tissues^[^ ^42^, ^43^, ^44^ ^]^ and human umbilical cord tissues,^[^ ^45^, ^46^ ^]^ in this study, we have successfully isolated and identified MSCs from human keloid tissues (KMSCs), which provided a reliable system to study cell fate conversion and intervention. Our results revealed functional heterogeneity within KMSCs: DPP4^−^ KMSCs possess increased adipogenic potential, while DPP4^+^ KMSCs favor fibrotic differentiation. These findings suggested that DPP4 acts not only as a surface marker but also as a functional molecule precisely linking fibrotic and adipogenic processes critical for scar pathogenesis. Additionally, DPP4 has many known inhibitors; of these, sitagliptin, the first DPP4 inhibitor to receive marketing approval with an inherent long half‐life,^[^ ^47^ ^]^ was selected based on the principle of “drug repurposing”. We demonstrated that sitagliptin induces cell fate transition from fibroblasts toward adipocytes in DPP4^+^ KMSCs, resulting in suppressed scar formation.

**Figure 7:**
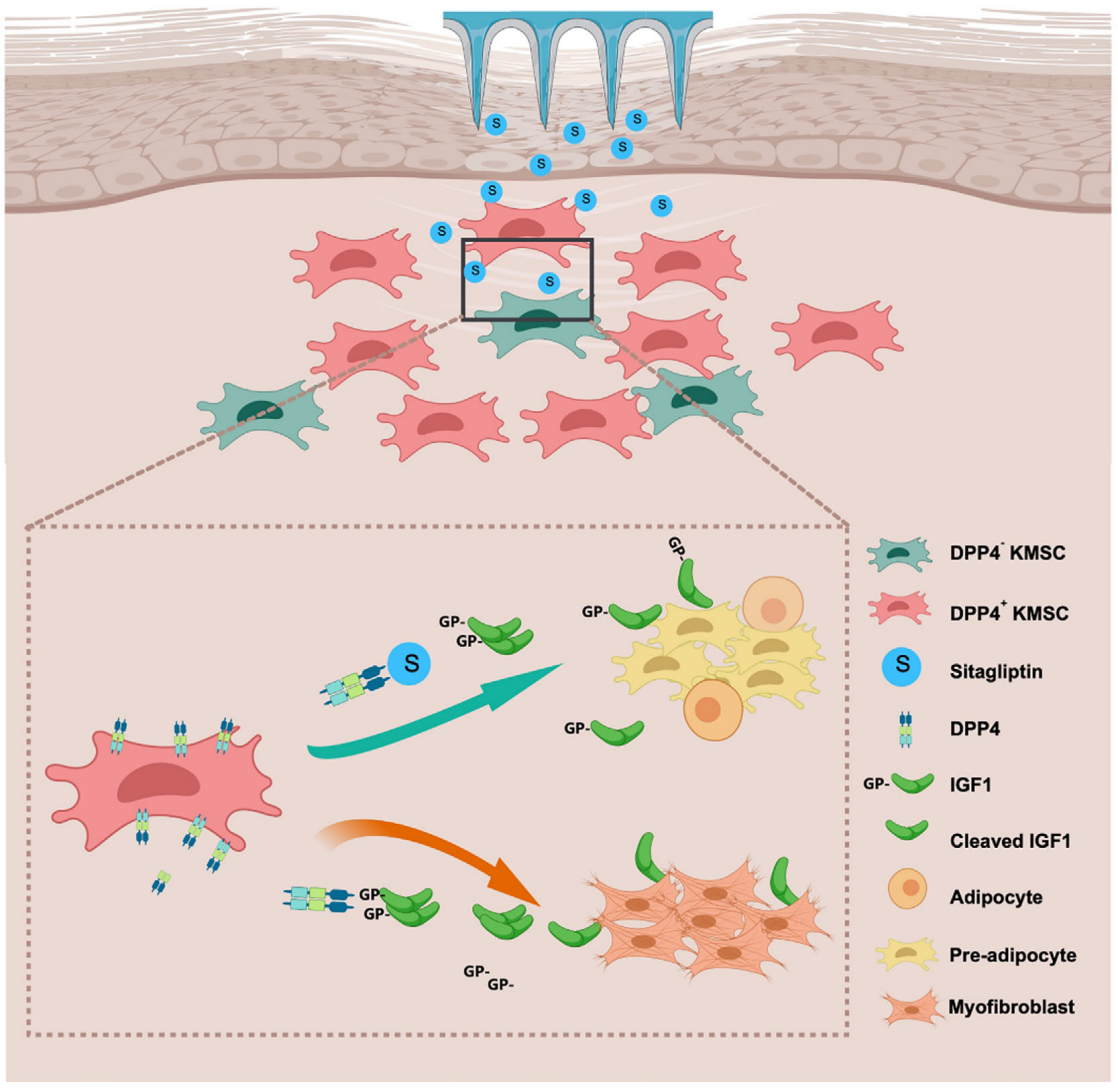
Illustration of our work. In scar tissues, IGF1 is cleaved by DPP4 enzyme in DPP4^+^ KMSC. Cleaved IGF1 may induce KMSC differentiation toward myofibroblasts, whereas IGF1 protected by sitagliptin acts as an adipogenic agent.

Mechanistically, we focused on the potential substrates of DPP4 associated with adipogenesis and selected BMP4 and IGF1 for further investigation. Data showed direct cleavage of IGF1 rather than BMP‐4 by DPP4. IGF1 exerts a stronger adipogenic induction than insulin,^[^ ^33^ ^]^ which has always been supplemented as a key cytokine in the adipogenic induction of MSCs. It is likely that elevated DPP4 levels in keloids inhibit the adipogenic capacity of IGF1, which can be restored by sitagliptin. Supporting our conclusions, a DPP4 inhibitor has been reported to restore the insulin responsiveness of adipocytes.^[^ ^48^ ^]^ A previous report suggested fibrogenic role of IGF1 in hypertrophic scarring, stimulating collagen secretion by fibroblasts.^[^ ^49^ ^]^ The possible explanation is that differential activities arise from truncated versus full‐length IGF1: cleaved IGF1 may promote myofibroblast differentiation in KMSCs, whereas intact IGF1 or sitagliptin‐protected IGF1 exerts a proadipogenic effect (**Figure** **7**). This difference between full‐length and truncated proteins is observed in several other DPP4 substrates, such as C‐X‐C motif chemokine 12 (CXCL12), interleukin‐3 (IL‐3), and granulocyte‐macrophage colony‐stimulating factor (GM‐CSF).^[^ ^20^, ^50^, ^51^ ^]^ Comprehensive proteomic screening could reveal additional novel regulatory targets beyond those identified in this study.

Sitagliptin is extensively used for type 2 diabetes treatment, exhibiting a low risk of hypoglycemia with minimal side effects through oral administration.^[^ ^21^, ^22^ ^]^ Nonetheless, concerns remain regarding oral sitagliptin administration for scar treatment, including insufficient local concentrations in the skin and treatment‐related risks for non‐diabetic individuals.^[^ ^52^ ^]^ To address these issues, we utilized the recently established^[^ ^36^ ^]^ hydrogel MNP system to deliver sitagliptin or IGF1 directly into the wound bed, achieving a sustained, localized therapy. By providing more direct spatial and temporal controls within the wound tissues, MNP system can not only avoid the potential risks of systemic drug administration to improve safety, but also provide a treatment strategy with painless penetration and higher patient compliance. Moreover, HAMA hydrogel MNPs create a favorable microenvironment for promoting wound healing and tissue repair and regeneration.^[^ ^53^ ^]^

In our dorsal wound model, MNP administration notably reduced scar formation and enhanced adipogenesis during murine wound healing, highlighting its suitability for early‐stage intervention. Notably, sitagliptin was released at a higher rate during the first hour and exhibited a less effective slow‐release profile in the subsequent period compared with that of the IGF1 group. Although sitagliptin proved significantly effective at inhibiting scarring and promoting cell fate conversion in vivo, further studies are needed to improve its release profile. Intriguingly, our study revealed that sitagliptin or IGF1 loaded MNPs exerted antiscarring effects without altering the wound closure rate. We proposed two possible reasons: First, while the intricate interplay between epidermal and dermal layers has been well documented in both clinical observations and experimental models, these findings suggest that dermal repair and epidermal regeneration may represent distinct biological processes during wound healing. Another reason is that DPP4 is predominantly expressed in dermal fibroblasts, with minimal expression detected in keratinocytes. This cell type‐specific expression may explain why sitagliptin—a DPP4 inhibitor—exerts its effects primarily within the dermal fibroblasts, thereby reducing scar formation without altering the epithelial regeneration process.

There are several limitations to this study. First, owing to the inherent differences in skin architecture and wound healing mechanisms between mice and humans, it is challenging to observe long‐term effects on wound healing and therapeutic effects on mature scarring. Therefore, dermal thickness, Masson staining, and α‐SMA expression were used to provide quantitative histopathological assessment of scar formation.^[^ ^12^ ^]^ Furthermore, during skin wound repair in mice, skin contraction contributes significantly to the reduction in wound size. For maintaining the scar formation, silicone rings were used to stabilize the skin and suppress contraction, as previously reported,^[^ ^7^, ^12^ ^]^ but this may have extended the healing timeline.

In this study, we have successfully isolated and characterized KMSCs, uncovering divergent adipogenic (DPP4^−^) and fibrogenic (DPP4^+^) potentials both in vitro and in vivo. Targeted inhibition of DPP4 activity with sitagliptin reprogrammed KMSC differentiation at least in part through IGF1 activity protection. Utilizing a MNPs‐based delivery platform, we demonstrated the capacity of locally administered sitagliptin or IGF1 to prevent scar formation in murine models. These findings establish a cell fate conversion approach as an effective anti‐scar strategy, with potential to improve the quality of skin wound healing.

This drug‐loaded MNPs represents a promising and innovative strategy with significant translational potential for scar prevention and treatment in clinical settings. Transdermal microneedle delivery systems have been extensively validated for their safety and efficacy in fields such as diabetes management and vaccination,^[^ ^54^ ^]^ demonstrating their adaptability and reliability in diverse patient populations, including pediatric and outpatient care scenarios. These prior successes provide a solid foundation for the rapid clinical translation of this technology.

There are several clinical advantages of this MNP system. First, it improves the patient compliance: A single‐application, minimally invasive therapy without the need for painful injections substantially alleviates treatment‐associated discomfort while enhancing accessibility, particularly for vulnerable populations such as pediatric patients. Besides, it allows early‐stage therapeutic intervention: in contrast to conventional postinjury scar management, this system allows early modulation of cellular behaviors, such as stem cell differentiation and fibroblast activity immediately following injury. This proactive mechanism aligns with regenerative medicine principles and represents a paradigm shift in wound care.

Nevertheless, important challenges persist for clinical translation and large‐scale manufacturing. It is critical to preserve nanoscale precision during microneedle mold fabrication to maintain bioactivity, and to implement stringent quality control for batch uniformity. Long‐term biocompatibility and safety assessments are also required to evaluate the degradation and potential byproducts in acute and chronic wound scenarios. Future studies employing large‐animal models, such as porcine skin, which closely approximates human physiology and structure, will further enhance translational relevance and support regulatory approval.

## 4 Conclusion

Taken together, the unique advantages of microneedle‐based transdermal drug delivery systems offer an exciting opportunity to redefine the clinical management of cutaneous wound healing. With advancements in manufacturing technology and rigorous biocompatibility research, this strategy holds great promise to enable “scarless healing”, and to address persistent challenges in regenerative medicine and wound care.

## 5 Experimental Section

5.1

5.1.1

##### Isolation and Culture of Keloid‐Derived Fibroblasts

Keloid samples were collected from patients who underwent keloid resection. The patient had no history of infection or prior treatment. Written informed consent was obtained from all patients. This study was approved by the Medical Ethics Committee of Chinese PLA General Hospital (S2023‐197‐01). Keloid‐derived fibroblasts were isolated and cultured using the modified explant culture method described by Li et al.^[^ ^26^ ^]^ Keloid tissues were initially placed in phosphate‐buffered saline (PBS) with penicillin (1000 U mL^−1^) and streptomycin (1000 μg mL^−1^) for 30 min (15 240 062, Gibco, Grand Island, NY). The epidermal tissues were carefully removed using sterile scissors. After several washes with PBS, the tissues were minced into 1.5 mm pieces, seeded in 25 cm^−^^2^ culture flasks, and cultured in low‐glucose Dulbecco's modified Eagle medium (DMEM, C11885500BT, Gibco) supplemented with 20% fetal bovine serum (FBS, 10099141C, Gibco), penicillin (100 U mL^−1^), and streptomycin (100 μg mL^−1^). The cultures were maintained in a 37 °C, 5% CO_2_, humidity‐saturated incubator. The medium was replaced every 2 days with medium containing 10% FBS. When the fibroblasts reached 80% confluence, they were digested with 0.25% trypsin (SLCJ1940; Sigma‐Aldrich, St. Louis, MO) for 2 min, neutralized with 10% FBS, collected, centrifuged, and cultured for further experiments. Sitagliptin (20 μM, S5079, Selleck, Houston, TX) was added for DPP4 inhibition. The concentration of sitagliptin was determined by its effect on the expression of PPARγ and α‐SMA, and evaluated using immunofluorescence. Recombinant human TGF‐β1 (10 ng mL^−1^, 100‐21, PeproTech, Rocky Hill, NJ) was added to simulate a fibrotic microenvironment as previously reported.^[^ ^30^ ^]^ RNA extraction was performed 24 h after TGF‐β1 and Sitagliptin treatment, and immunofluorescence staining was conducted 72 h post‐treatment. PPP (50 nM, S7668, Selleck) was added for IGF‐1R inhibition, and the dose used was determined by both the previous study^[^ ^55^ ^]^ and our pretest study.

##### Identification of Protein Truncation by DPP4

To identify the direct truncation of IGF1 by DPP4, recombinant human IGF1 (rhIGF1, 291‐G1, R&D Systems, Minneapolis, MN) was incubated with a recombinant human DPP4 Fc chimera (11141‐SE, R&D Systems). rhDPP4/Fc was diluted to a concentration of 0.2 ng μL^−1^ in the assay buffer, and 1 μL of the diluted rhDPP4/Fc was added to 15 μL of diluted rhIGF1 (1 μg μL^−1^). The mixture was allowed to react for 30 min at room temperature. For the negative control, 5 μL of rhIGF1 was added to the solution before quick‐freezing with liquid nitrogen and storage at −80 °C. HPLC‐MS/MS (Qinglian Biotech, Beijing, China) was used for analyzing the reaction. For mass spectrometry analysis, an ORBITRAP ECLIPSE mass spectrometer equipped with a FAIMS Pro interface was used. Proteome Discoverer 2.4 software was used for the database search.

##### Preparation of Hydrogel MNPs

The HAMA‐gel precursor solution was prepared using 5% w/v HAMA (EFL‐HAMA‐150 K, Engineering For Life, Suzhou, China) and 0.25% w/v lithium phenyl‐2,4,6‐trimethylbenzoylphosphinate (LAP, Engineering For Life). Sitagliptin phosphate monohydrate (2 mg mL^−1^; S4002, Selleck) and IGF1 (500 ng mL^−1^; 291‐G1; R&D Systems,) were dissolved in the precursor solutions. The precursor solution was added to a mold (Microneedle height: 500 μm, base diameter: 270 μm, needle spacing: 700 μm) and dried for 12 h at 30 °C for condensation, in which the IGF1 bioactivity remains unaffected according to Clark et al. report.^[^ ^56^ ^]^ Then, it was followed by photocuring for 30 s with UV405 at an intensity of 30 mW cm^−^^2^. The base of the MNPs was prepared with PVA (20%; Engineering For Life) and again dried for 12 h. The structure of the MNPs was observed using a stereomicroscope and an SEM (GeminiSEM 300, ZEISS, Oberkochen, Germany). The compressive strength of HAMA MNPs was determined using a universal testing machine (Zhongluchang, Jinan, China).

##### Swelling Assay

For the swelling assay, the patches were weighted (*W*_0_), and then soaked in 5 mL PBS. Then the patches were weighted (*W*_t_) at several time points. Swelling ratio was calculated using the following formula:

(1) $$Swelling\;Ratio=\left(W_{t}-W_{0}\right)/W_{0}\times 100\%$$

##### Transplantation of Keloid‐Derived Fibroblasts

For cell transplantation, sorted DPP4^+/−^ fibroblast suspensions were mixed with Matrigel (354 262, Corning, NY) at a 1:1 ratio to achieve a final concentration of 8 × 10^6^ cells per 200 μL. The resulting cell suspensions were subcutaneously injected into the right axillary region of 6‐week‐old BALB/c nude mice (Vital River Laboratory Animal Technology, Beijing, China), with 200 μL per injection. Following transplantation, the implant growth was monitored and measured every three days. After one month, the mice were euthanized with an overdose of pentobarbital sodium (200 mg kg^−1^, i.p.), and the transplanted keloid‐like structures were harvested for further analysis.

##### Cutaneous Scar Model

To establish a cutaneous scar model in mice, female C57BL/6 J mice aged 6–8 weeks were randomly divided into four groups. A dose of 1% pentobarbital sodium (40 mg kg^−1^) was intraperitoneally injected for sedation. The dorsal hair was shaved, and a depilatory cream was applied for complete hair removal. Full‐thickness skin defects were created in the dorsal region using a 6 mm diameter skin punch (day 0). A silicone ring with a diameter of 10 mm was sutured around the wound to prevent contraction. The wound was photographed, and MNPs or routine treatment were performed in the wounds every three days. Eighteen days later, the mice were sacrificed, and the tissues of the wound area were harvested for further analysis. For quantitative analysis, the unhealing rate was determined by calculating the percentage of unhealed wound size relative to the original wound size ([residual area]/[baseline area] × 100%). All animal procedures were approved by the Institutional Animal Care and Use Committee and complied with the guidelines of the Beijing Medical Experimental Animal Care Commission (IACUC‐DWZX‐2023−533).

##### Skin Puncture Experiment

To perform skin puncture experiments, C57BL/6J mice aged 6–8 weeks were anesthetized with 1% pentobarbital sodium (40 mg kg^−1^). Dorsal hair was shaved, and a depilatory cream was applied for complete hair removal. The MNPs were then pressed onto the bald skin and removed after 1 min. Photographs of the skin were taken both before and after the puncture, and the used patch was examined and photographed using a stereomicroscope (SZX9‐3132, Olympus, Tokyo, Japan).

##### Histopathological Examination

The obtained tissues were immediately fixed in 4% paraformaldehyde for 24 h and embedded in paraffin. The tissues were cut into 5 μm‐thick sections, deparaffinized, and hydrated for further examination. For H&E staining, the sections were stained with hematoxylin for 5 min to stain the nuclei, followed by rinsing. The sections were then subjected to bluing for enhanced nuclear staining. Eosin was applied for 3 min, followed by a brief rinse in 95% ethanol to remove the excess stain. Finally, the sections were dehydrated and mounted with neutral gum for microscopic examination. Dermal thickness was quantified in H&E‐stained tissue sections using Image J software. For immunofluorescence staining, hydrated sections were incubated with an antigen repair solution according to the antibody requirements. The sections were then permeabilized and blocked before incubation with the antibodies listed in Table S1, Supporting Information. After staining, the sections were observed and photographed using TissueFAX PLUS (TISSUE GNOSTICS, Vienna, Austria). Photographs of five random fields were used to quantitatively analyze the staining using Image J software (version 1.54f, USA). Quantification of α‐SMA protein expression was conducted by measuring fluorescence intensity, while adipogenic differentiation was evaluated through enumeration of PPARγ‐ and C/EBPα‐positive cells. Masson staining was performed using a Masson staining kit (G1006, Servicebio, Wuhan, China) according to the manufacturer's instructions. The blue‐stained regions of Masson staining or positively stained regions of COL1A1 were quantified using Image J software to calculate the percentage area of collagen deposition.

##### Statistics Analysis

Data were analyzed using SPSS 26.0. No preprocessing of data was performed. Student's *t*‐test was used to compare data between two groups, and one‐way analysis of variance (ANOVA) followed by Tukey's HSD post hoc test was applied for multiple group comparisons. Statistical significance was set at *p* < 0.05. Data were presented as mean ± SEM. Sample sizes for each statistical analysis were provided in the figure legend.

## Supporting Information

Supporting Information is available from the Wiley Online Library or from the author.

## Conflict of Interest

The authors declare no conflict of interest.

## Supporting information

Supplementary Material

## Data Availability

The data that support the findings of this study may be found in a data supplement available with the online version of this article or from the corresponding authors upon reasonable request.
