# Supplementary material for: Sustained‐Release Sitagliptin Microneedles for Scar Prevention via Fibroblast‐to‐Adipocyte Conversion
Source: Small Sci. 2025 Oct 21;5(12):e202500140. doi: 10.1002/smsc.202500140 (PMC12697911; doi:10.1002/smsc.202500140)
Supplement: Supplementary file 1 — Supplementary Material [file SMSC-5-e202500140-s001.pdf]

Supporting Information

Sustained-release Sitagliptin Microneedles for Scar Prevention via Fibroblast-to-Adipocyte Conversion

*Ju-Lei Zhang [#], Jun-Nian Zhou [#, *], Chao Tang [#], Yan Li, Wen-De Yao, Ling-Li Guo, Zhao-Yang Chen, Ya-Li Jia, Quan Zeng, Biao Zhang, Tao Fan, Jia-Fei Xi, Xue-Tao Pei, Yan Han[*], Wen Yue [*]*

J. L. Zhang, W.D. Yao, L.L. Guo, Z.Y. Chen, Y. Han

Department, University, City, Country

Department of Plastic and Reconstructive Surgery, The First Medical Centre, Chinese PLA General Hospital, 28 Fuxing Street, Beijing 100853, China

E-mail: 13720086335@163.com (Y. Han)

J. L. Zhang, J.N. Zhou, C. Tang, Y. Li, W.D. Yao, Z.Y. Chen, Y.L. Jia, Q. Zeng, B. Zhang, T. Fan, J.F. Xi, X.T. Pei, W. Yue

Beijing Institute of Radiation Medicine, 27 Taiping Road, Beijing 100850, China

E-mails: zhoujunnian@bmi.ac.cn (J.N. Zhou), yuewen@bmi.ac.cn (W. Yue).

Y. Li, Y. Han

Department of Plastic Surgery, Peking University International Hospital, 1 Shengmingyuan Road, Beijing 102206, China

# Ju-Lei Zhang, Jun-Nian Zhou, and Chao Tang contributed equally to this work

* Corresponding authors

Supplementary Methods

Characterization of multi-lineage differentiation potential of keloid-derived fibroblasts

For osteogenic differentiation, OriCell® human adipose mesenchymal stem cell osteogenic differentiation medium (HUXMD-90021, Oricell, USA) was used according to the manufacturer's protocol. Briefly, each well of a six-well plate was coated with 1 mL of 0.1% gelatin and placed in a CO_2 incubator. After 30 min, the gelatin was removed and keloid-derived fibroblasts were seeded and cultured in a six-well plate at 2×10^4 cells/cm^2. Once the cell confluence reached 70%, the medium was replaced with 2 mL of osteogenic induction medium and refreshed every three days over two weeks. To observe calcium nodules, the cells were washed 2–3 times with 1×PBS and fixed with 2 mL of 4% paraformaldehyde solution (P1110, Solarbio, China) at room temperature for 30 min, followed by staining with 2 mL of Alizarin Red working solution at room temperature for 5–10 min. The excess dye was washed away with 1×PBS. Plates were observed and photographed under a microscope (CKX31; Olympus, Japan). The images were analyzed using ImageJ (1.54f, USA) to evaluate the stained areas.

For chondrogenic differentiation, OriCell® human adipose mesenchymal stem cell

chondrogenic differentiation medium (HUXMD-90041, Oricell) was used according to the manufacturer's protocol. Briefly, 4×10^5 fibroblasts were transferred to a 15 mL centrifuge tube and centrifuged at 250×g for 4 min. The supernatant was removed and chondrogenic induction premix (0.5 mL) was added to resuspend the pellet, followed by two additional rounds of centrifugation at 150×g for 5 min. The cells were then resuspended in complete chondrogenic induction medium (0.5 mL) and centrifuged at 150×g for 5 min. After loosening the cap of the centrifuge tube, it was placed in a 37°C incubator with 5% CO_2 and saturated humidity for 24 h without agitation. The medium was refreshed every 2–3 d with complete chondrogenic induction medium. Continuous induction was performed until spheroids approximately 1.5–2 mm in diameter were formed within approximately 2 weeks). For histological examination, cartilage spheroids were washed with 1×PBS and fixed in 4% paraformaldehyde. To observe acid mucopolysaccharides, the fixed cartilage spheroids were embedded in paraffin and stained with Alcian blue. The sections were observed and photographed using TissueFAX PLUS (TISSUE GNOSTICS, Austria). The images were analyzed using ImageJ to evaluate the stained area.

For adipogenic differentiation, OriCell® human adipose mesenchymal stem cell adipogenic differentiation medium (HUXMD-90031, Oricell) was used according to the manufacturer's protocol. Briefly, 0.1% gelatin was added to six-well plates and

incubated for 30 min. Keloid-derived fibroblasts were seeded at a density of 2×10^4 cells/cm^2 in each well, cultured with DMEM at 37°C with 5% CO_2. Once the cells reached 100% confluence, the medium was replaced with 2 mL adipogenic induction medium A and maintained for 3 d followed by replacement with 2 mL of medium B for 1 d. Medium B was then and replaced again with medium A to continue the induction process. This process was repeated until a sufficient number of lipid droplets were observed. For Oil Red O staining, the induction medium was removed, the cells were gently washed 2–3 times with 1×PBS, and fixed in 2 mL of 4% paraformaldehyde solution for 30 min. The wells were washed and stained with 2 mL of Oil Red O working solution at room temperature for 30 min. The cells were washed again, examined, and photographed under a microscope (CKX31; Olympus, Japan). The images were analyzed using Image J.

In differentiation-induction experiments, total RNA was harvested on day 3 of induction for subsequent analyses.

Flow cytometry analysis

Keloid-derived fibroblasts were enzymatically digested, collected, and resuspended in 100 μL of PBS. They were then incubated with the indicated antibody for 30 min at 4°C, protected from light. The antibodies used in this study are listed in

Supplementary Table 1. After incubation, the cell suspensions were washed with PBS and centrifuged to collect the stained cells, which were resuspended in PBS and passed through a 40 μm strainer. The cells were analyzed using a BD FACSAria flow cytometer (BD Biosciences, Franklin Lakes, NJ, USA) and FlowJo software (TreeStar, Ashland, OR, USA).

Colony-forming unit for fibroblasts (CFU-F) assay

Sorted DPP4$^{+/-}$ cells were seeded into a six-well plate at 1000 cells/well in 2 mL of DMEM low-glucose medium plus 10% FBS; the medium was replaced every seven days. After two weeks of culture, colony formation was observed, the cells were fixed with 4% formaldehyde for 30 min, and stained with 0.1% crystal violet (C0121, Beyotime, China) for 3 min. Images of the stained colonies were obtained using a stereo microscope.

Real-time quantitative PCR

RNA was extracted from the cells using the RNeasy Extraction Kit (Qiagen, Hilden, Germany). cDNA was obtained by reverse transcription using ReverTra Ace (TOYOBO, Osaka, Japan). Real-time quantitative PCR was performed using CFX ConnectTM (Bio-Rad, USA) and THUNDERBIRD Nxt SYBR qPCR Mix (QPX-

201; TOYOBO, Japan). The primers used are listed in Supplementary Table 2.

Western blotting

IGF1 (10 ng/mL, 291-G1, R&D System, USA) or picropodophyllin (50 nM, S7668, Selleck, USA), was added during adipogenic induction of keloid-derived fibroblasts. Samples were collected on day 7 for analyzing indicated protein expression levels. Proteins were obtained using RIPA Lysis Buffer (P0013B, Beyotime, China), separated by molecular weight using SDS-PAGE, and transferred to polyvinylidene fluoride membranes. After transfer, the membranes were blocked with non-fat milk (5%) and incubated overnight at 4°C with primary antibodies. An HRP-conjugated secondary antibody was added after washing the membrane. Chemiluminescence analysis was performed to detect protein expression levels. An Amersham Imager 680 (GE, USA) was used to visualize the images. Data were analyzed using Image J software. The antibodies used in this study are listed in Supplementary Table 1.

Release experiment of sitagliptin and IGF1

The precursor solution of HAMA-gel loaded with sitagliptin or IGF1 was added into a 24-well plate and photocured, with 100 μL per well. PBS was added to the wells at 800 μL/well. The plates were then incubated at 37°C. At 1, 3, 6, 12, 24, 48, and 72 h, 400 μL of the extraction solution was removed for measuring the released sitagliptin

or IGF1, and another 400 μL PBS was added and mixed gently. The percentage of released reagents was calculated. IGF1 and sitagliptin were detected using a BCA Protein Assay Kit (23227, Thermo Fisher, USA) and a spectrophotometer (NanoDrop 2000c, ABI, USA, 263 nm), respectively.

***In vitro* biocompatibility testing**

The precursor solution of HAMA-gel loaded with sitagliptin and IGF1 was added to a 6-well plate and photocured to form a gel, with 400 μL/well. An extraction solution was prepared by adding 1 mL of DMEM containing 10% FBS to immerse the photocured gel. The mixture was incubated at 37°C for 72 h. After incubation, the solution was passed through a 0.22 μm filter to obtain the extraction solution. Keloid-derived fibroblasts were cultured in a 6-well plate. After 24 h of culturing, the cells were treated with 2 mL of the extraction solution or control DMEM medium for further 24 h. The cells were then subjected to live/dead staining using a Calcein AM/PI staining kit (C2015S, Beyotime, China) according to the manufacturer's instructions. Briefly, the medium was removed and cells were washed gently with PBS for 1 time, then 1 ml Calcein AM/PI work solution was added into the wells and incubated in dark for 30 min. Then the stained cells were observed and photographed using a fluorescence microscope.

Mass Spectrometry Analysis

Protein was mixed with 5 mM DTT (final concentration) and incubated at 37°C for 1 hour, followed by cooling to room temperature. Lodoacetamide was added to a final concentration of 10 mM, and the mixture was incubated in the dark at room temperature for 45 minutes. The sample was diluted 4-fold with 25 mM ammonium bicarbonate, and trypsin was added at a protein-to-trypsin ratio of 50:1. The mixture was incubated overnight at 37°C. The next day, formic acid was added to adjust the pH to below 3 to terminate digestion.

The sample was desalted using a C18 desalting column. The column was activated with 100% acetonitrile, equilibrated with 0.1% formic acid, and loaded with the sample. Impurities were washed away using 0.1% formic acid, and the sample was eluted with 70% acetonitrile. The eluent was collected and lyophilized.

Mobile phases A (100% water, 0.1% formic acid) and B (80% acetonitrile, 0.1% formic acid) were prepared. The lyophilized powder was dissolved in 10 μL of mobile phase A, centrifuged at 14,000 × g for 20 minutes at 4°C, and 1 μg of the supernatant was injected for LC-MS analysis. An ORBITRAP ECLIPSE mass spectrometer equipped with a FAIMS Pro™ Interface was used. The compensation voltage (CV) alternated between -45 and -65 every 1 second. The Nanospray Flex™ (NSI) ion

source was operated at a spray voltage of 2.0 kV, with an ion transfer tube temperature of 320°C. Data-dependent acquisition (DDA) mode was employed for full MS scans over a range of m/z 350–1500. The primary MS resolution was set to 120,000 (at m/z 200), with an automatic gain control (AGC) target of 4×10^5 and a maximum injection time of 50 ms. For MS/MS, the "Top Speed" mode was used with a secondary resolution of 15,000 (at m/z 200), an AGC target of 5×10^4, a maximum injection time of 22 ms, and a collision energy of 33%. Raw data files (.raw) were generated for subsequent analysis. The database search was performed using Proteome Discoverer 2.4 software with the following parameter settings: Enzyme, Trypsin, Glu-C; Static Modification, Carbamidomethyl (C); Dynamic Modification, Oxidation (M; 15.995 Da), Acetyl (Protein N-terminal); Precursor ion mass tolerance, ±15 ppm; Fragment ion mass tolerance, ±0.02 Da; Max Missed Cleavages, 2.

IC_{50} and CCK8 assay

Cells were cultured with series of concentration of TGF-β1 or sitagliptin on 96-well plate, then the medium was removed, the cells were washed with PBS for 3 times. Then 100 μl of CCK-8 reagent was added into the well to incubate for 1 h before the absorbance (450 nm) was measured.

Supplementary Figures

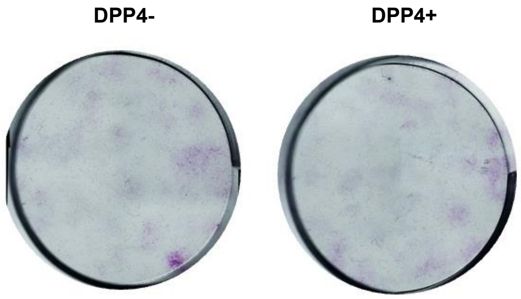

Supplementary Figure 1. Colony-forming unit for fibroblasts (CFU-F) assay. CFU-F assay was performed with sorted DPP4$^{+/-}$ cells to evaluate the clonogenic capacity.

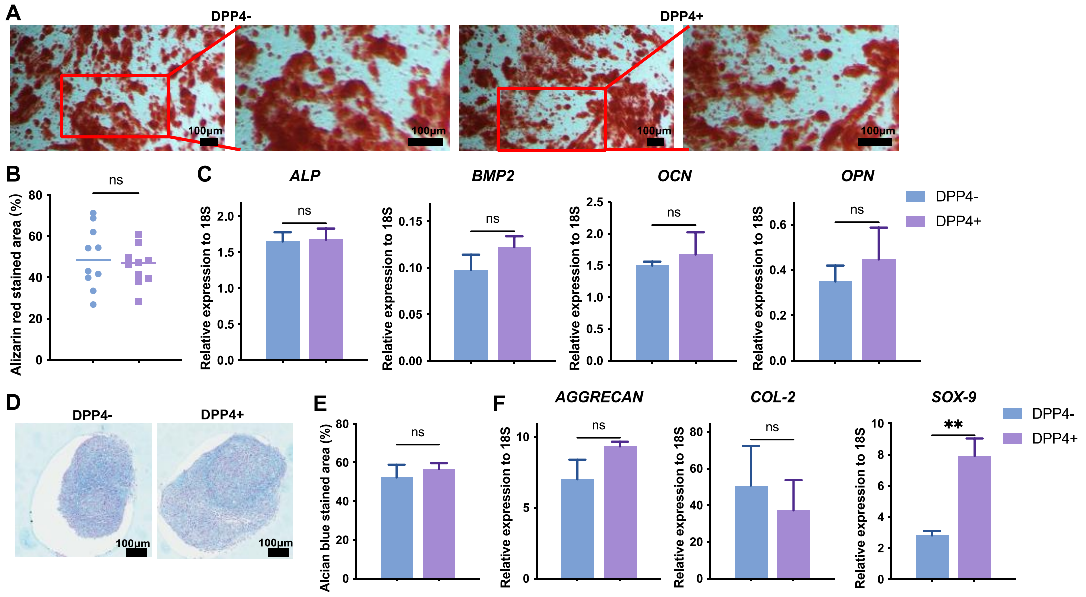

Supplementary Figure 2. Evaluation of osteogenic and chondrogenic potential of KMSC. (**A-C**) Sorted DPP4$^{+/-}$ fibroblasts were induced with osteogenic induction medium, the formation of calcium-rich mineralized matrix was evaluated with Alizarin red staining (**A-B**). n=10. The mRNA levels of osteogenic related genes were detected with RT-qPCR (**C**). n=3. **D-F**, sorted DPP4$^{+/-}$ fibroblasts were induced with chondrogenic induction medium, the acidic mucopolysaccharides formation was evaluated with Alcian blue staining (**D-E**). n=3. The mRNA levels of chondrogenic related genes were detected with RT-qPCR (**F**). n=3. *, *p*<0.05, **, *p*<0.01. Scale bars, 100 μm in **A, D**. Data was presented as mean ± SEM. Statistical significance was determined by Student's t-test.

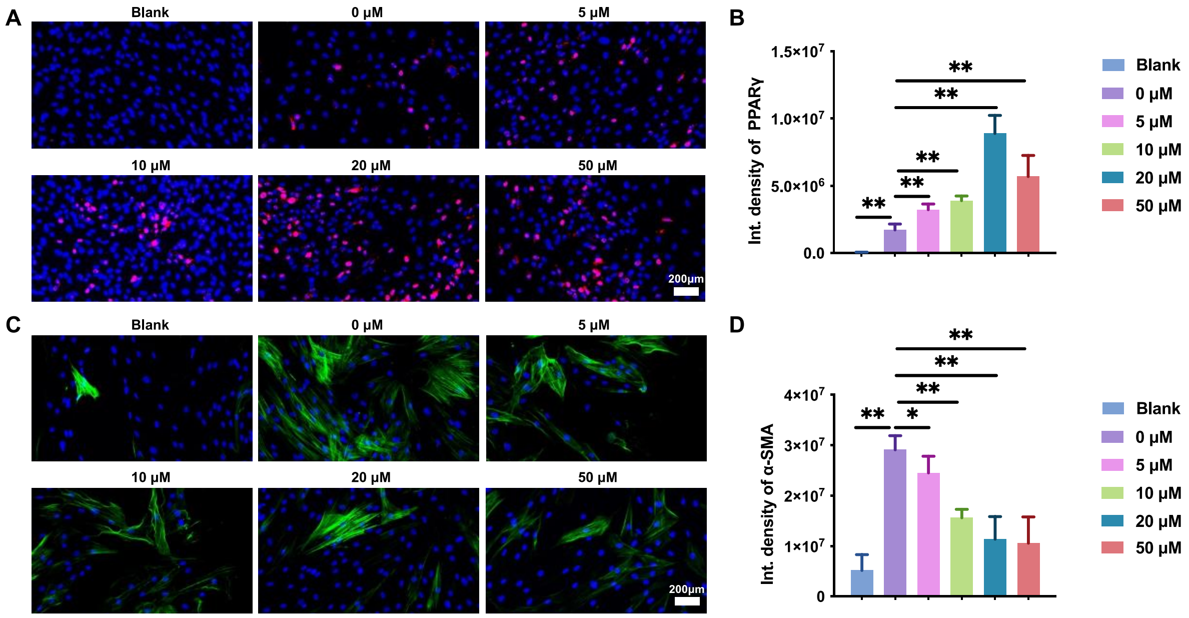

Supplementary Figure 3. The effect of sitagliptin with different concentration. (A-B) KMSCs were induced with adipogenic induction medium (except for Blank group) and treated with sitagliptin (0-100 μM), and the expression of PPARγ was evaluated by immunofluorescence staining after 7 days stimulation. n=5. **(A-B)** KMSCs were treated with TGF-β1 (except for Blank group) and sitagliptin (0-100 μM), and the expression of α-SMA was evaluated by immunofluorescence staining after 72 h stimulation. n=5. *, $p<0.05$, **, $p<0.01$. Scale bars, 200 μm. Data was presented as mean ± SEM. Statistical significance was determined by one-way analysis of variance (ANOVA) followed by Tukey's HSD post hoc test.

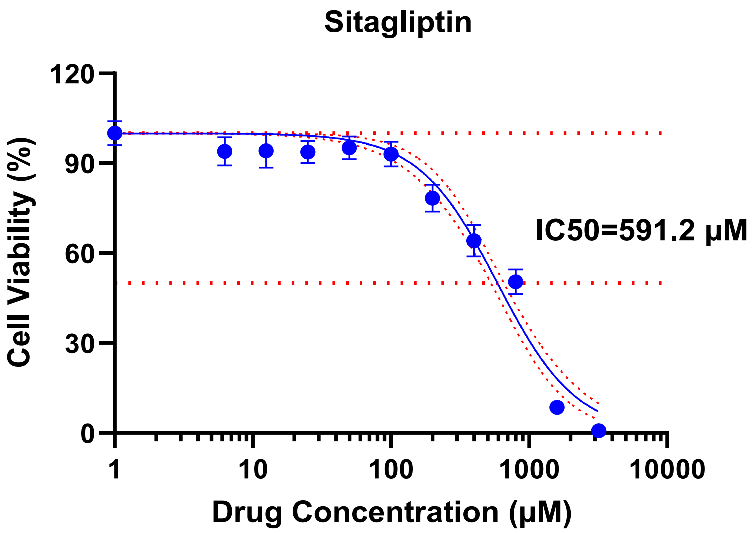

Supplementary Figure 4. Dose-response curve of keloid-derived stem cells treated with sitagliptin. KMSCs were treated with sitagliptin with different concentration for 48 h and the CCK8 assay was performed to detected the cell viability, and the IC_{50} of sitagliptin was calculated to be 591.2 μM. n=5.

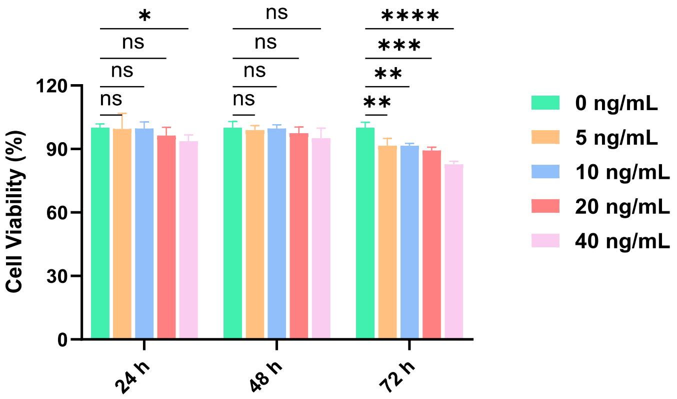

Supplementary Figure 5. Dose-/time dependent TGF-β effects on KMSC viability was evaluated with CCK8 assay. KMSCs were treated with TGF-β1 (0-40 ng/ml), and the CCK8 assay was performed to detected the cell viability. n=6. **p* < 0.05, ***p* < 0.01. ns, no significant. Data was presented as mean ± SEM. Statistical significance was determined by one-way analysis of variance (ANOVA) followed by Tukey's HSD post hoc test.

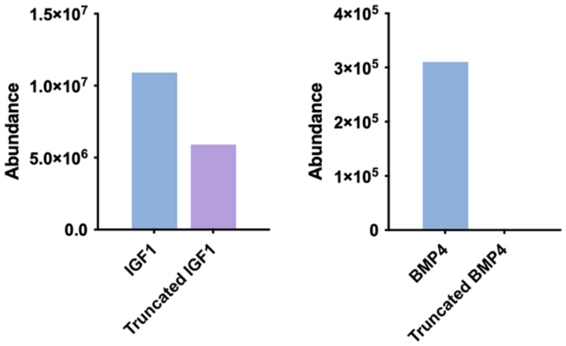

Supplementary Figure 6. Identification of the truncated product of IGF1/BMP4 by DPP4 Fc chimera. Recombinant human IGF1 or BMP4 was incubated with recombinant human DPP4 Fc chimera for cleaving assay, and the product was analyzed by HPLC-MS/MS. The peptide sequences were identified, and the abundance of truncated product from the cleaving assay were calculated.

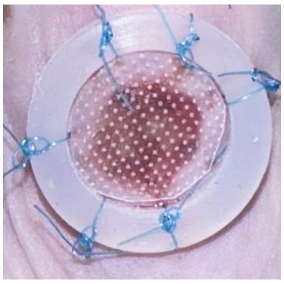

Supplementary Figure 7. The microneedle patch was inserted into the mouse wound tissues.

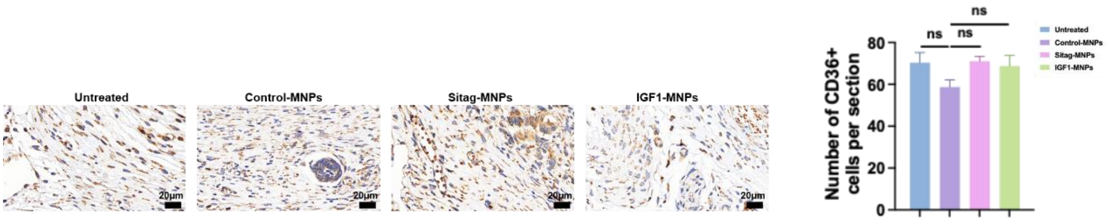

Supplementary Figure 8. Immunohistochemistry staining of CD36 and analysis related to Figure 6. n=6. ns, no significance. Scale bars, 20 μm. Data was presented as mean ± SEM. Statistical significance was determined by one-way analysis of variance (ANOVA) followed by Tukey's HSD post hoc test.

Supplementary Table 1. The antibodies used in the study.

Antibody	Applications	Cat. No./Source	Origin	Dilution
PPARγ	WB<br>IF	2435, CST	Rabbit	1:1000<br>1:100
CEBPα	WB<br>IF	8178, CST	Rabbit	1:1000<br>1:100
GAPDH	WB	2118, CST	Rabbit	1:1000
PERILIPIN1	IF	ab61682, Abcam	Goat	1:200
HLA-ABC	IF	ab225636, Abcam	Rabbit	1:4000
COL1A1	IF	72026, CST	Rabbit	1:200
α-SMA	IF	19245, CST	Rabbit	1:400
APC-anti-CD29	FC	561794, BD Pharmingen™	Mouse	10 μL/test
APC-anti-CD73	FC	560847, BD Pharmingen™	Mouse	5 μL/test
PE-cy7-anti-CD90	FC	25-0902-82, eBioscience	Mouse	0.06 μg/test
PE-anti-CD105	FC	568553, BD Pharmingen™	Mouse	5 μL/test
PE-anti-CD166	FC	560903, BD Pharmingen™	Mouse	5 μL/test

APC-anti-CD34	FC	555824, BD Pharmingen™	Mouse	5 µL/test
PE-anti-CD38	FC	555460, BD Pharmingen™	Mouse	10 µL/test
APC-anti-CD45	FC	561864, BD Pharmingen™	Mouse	5 µL/test
APC-anti-CD14	FC	561383, BD Pharmingen™	Mouse	5 µL/test
APC-anti-HLA-DR	FC	559868, BD Pharmingen™	Mouse	10 µL/test
APC-anti-CD31	FC	17-0311-82, eBioscience	Mouse	0.5 µg/test
APC-anti-CD26	FC	563670, BD Pharmingen™	Mouse	5 µL/test
Anti-Adiponectin	WB<br>IHC-P	ab317847, Abcam	Rabbit	1:1000<br>1:8000
Anti-TGF β1	IHC-P	GB11179, Servicebio	Rabbit	1: 200
Anti-CD36	IHC-P	GB112562, Servicebio	Rabbit	1: 600

Supplementary Table 2. The primers used in the study.

Gene Name	Forward primer (5'-3')	Reverse primer (5'-3')
CEBPα	GGTGCGTCTAAGATGAGGGG	GCATTGGAGCGGTGAGTTTG
CEBPβ	CTTCAGCCCGTACCTGGAG	GGAGAGGAAGTCGTGGTGC

PPARγ	ACCAAAGTGCAATCAAAGTGG	ATGAGGGAGTTGGAAGGCTCT
ADIPONECTIN	AACATGCCCATTCGCTTTACC	TAGGCAAAGTAGTACAGCCCA
ALP	TACAAGCACTCCCACTTCATC	AGACCCAATAGGTAGTCCACAT
BMP2	GAAGAACTACCAGAAACGAGTG	GGTGATGGAAACTGCTATTG
OCN	CTGTGACGAGTTGGCTGAC	AGCAGAGCGACACCCTAGA
OPN	CATTCCGATGTGATTGATAGTC	CTTCCTTACTTTTGGGGTCTAC
Aggrecan	GTGCCTATCAGGACAAGGTCT	GATGCCTTTCACCACGACTTC
COL-2	TGGACGCCATGAAGGTTTTCT	TGGGAGCCAGATTGTCATCTC
SOX-9	AGCGAACGCACATCAAGAC	CTGTAGGCGATCTGTTGGGG
COL1A1	GAGGGCCAAGACGAAGACATC	CAGATCACGTCATCGCACAAC
COL3A1	GGAGCTGGCTACTTCTCGC	GGGAACATCCTCCTTCAACAG
α-SMA	AAAAGACAGCTACGTGGGTGA	GCCATGTTCTATCGGGTACTTC
FN1	CGGTGGCTGTCAGTCAAAG	AAACCTCGGCTTCCTCCATAA
18s RNA	AACCCGTTGAACCCCATT	CCATCCAATCGGTAGTAGCG
Nanog	ACAACTGGCCGAAGAATAGCA	GGTTCCCAGTCGGGTTCAC
Oct4	CCTCACTTCACTGCACTGTA	CAGGTTTTCTTTCCCTAGCT

Sox2	CCCAGCAGACTTCACATGT	CCTCCCATTTCCCTCGTTTT
MYC	GGCTCCTGGCAAAAGGTCA	CTGCGTAGTTGTGCTGATGT
